# Rab11a-dependent recycling of Glut3 inhibits seizure-induced neuronal disulfidptosis by alleviating glucose deficiency

**DOI:** 10.1186/s13578-025-01396-9

**Published:** 2025-05-28

**Authors:** Sijun Li, Junrui He, Huimin Kuang, Xiaojuan Wang, Muhua Zhou, Dongmei Li, Baoren Kang, Honghu He, Lina He, Wei Lin, Yuan Lv

**Affiliations:** 1 Department of Geriatric rehabilitation/Clinical Research Center for Geriatric Disorders of Guangxi Zhuang Autonomous Region, Jiangbin Hospital of Guangxi Zhuang Autonomous Region, No 85 Hedi Road, Nanning, 530021 Guangxi Zhuang Autonomous Region China; 2 Department of Neurology, Jiangbin Hospital of Guangxi Zhuang Autonomous Region, No 85 Hedi Road, Guangxi Zhuang Autonomous Region, Nanning, 530021 China

**Keywords:** Seizures, Neurons, Glucose, Recycling, Disulfidptosis

## Abstract

Seizures can trigger neuronal glucose deficiency, thereby inducing disulfidptosis. Disulfidptosis is a novel cell death mechanism characterized by the abnormal accumulation of disulfide caused by glucose deficiency. However, the mechanism underlying disulfidptosis caused by glucose deficiency in seizures remains elusive. Rab11a-dependent recycling of glucose transporter 3 (Glut3) is closely related to glucose metabolism in neurons, which may contribute to neuronal disulfidptosis after seizures by abnormal glucose metabolism. So here we introduced a well-established in vitro model of seizures to evaluate cell survival, glucose levels, disulfidptosis biomarkers, Glut3 and Rab11a expression, the recycling ratio of Glut3, and the protein complex of Glut3-Rab11a. Cell survival rates and glucose levels were lower in the in vitro model of seizures, accompanied by elevated levels of disulfidptosis markers. Moreover, the surface expression and the recycling ratio of Glut3, as well as the protein complex of Glut3-Rab11a, were positively correlated with Rab11a expression. Lastly, Rab11 overexpression improved cell survival rates, increased glucose levels, and decreased the levels of disulfidptosis biomarkers in the in vitro model of seizure. Rab11a-dependent recycling of Glut3 inhibited seizure-induced neuronal disulfidptosis by alleviating glucose deficiency.

**Supplementary Information:**

The online version contains supplementary material available at 10.1186/s13578-025-01396-9.

## Graphical Abstract

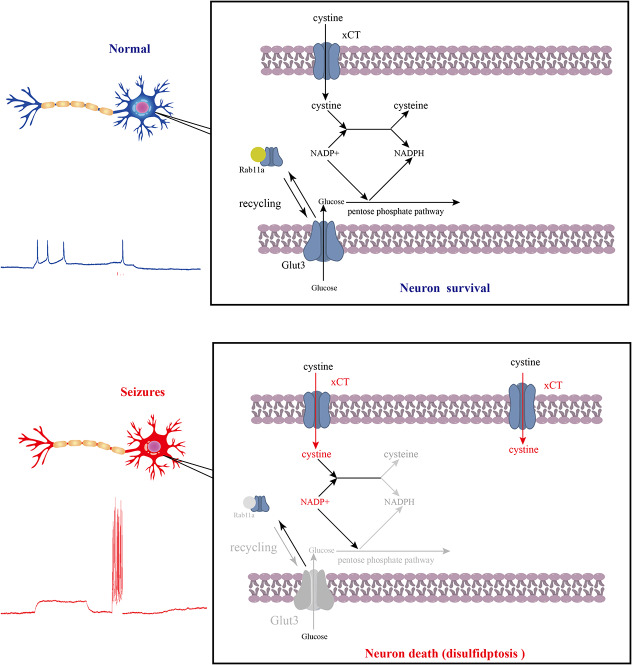

## Introduction

As is well documented, epilepsy (EP) is a complex disease caused by the synchronous discharge of neurons, with seizures as the primary manifestation [1]. Seizures are predominantly triggered by increased excitatory function and/or decreased inhibitory function of neurons, a mechanism also referred to as excitatory/inhibitory imbalance (E/I imbalance) [2–4]. However, E/I imbalance is not the only potential mechanism for seizures. Previous studies demonstrated that glucose metabolism disorders in the brain may also be involved in the occurrence and progression of seizures [5–8]. Hildebrand et al. described that lower glucose levels in the brain may be a key cause of seizures [9]. Longer durations of seizures are linked to lower glucose metabolism in the hippocampus on the ipsilateral side of the epileptic focus [10]. Moreover, low glucose metabolism in the brain may be linked to Sudden Unexpected Death in Epilepsy (SUDEP) [11]. Taken together, these studies suggest that seizures are associated with reduced glucose levels in neurons, which is likely to trigger disulfidptosis.

Disulfidptosis is considered a novel cell death mechanism characterized by the abnormal accumulation of cystine (Cyss), a type of disulfide with high intracellular cytotoxicity, that eventually results in cell death induced by disulfide stress [12]. Under physiological conditions, glucose generates a reduced form of nicotinamide adenine dinucleotide phosphate (NADPH) via the pentose phosphate pathway, which provides the reducing power to counteract the toxic effects of disulfide stress on cells. Under glucose-deficient conditions, the excessive Cyss uptake and Cyss reduction to cysteine (Cys) deplete the NADPH pool, leading to an imbalance in the NADP+/NADPH ratio, the accumulation of disulfides, and eventually rapid cell death [13–16]. During this process, members of the solute carrier family, SLC7A11 and SLC3A2, form complexes that facilitate cystine transport and accumulation into cells [17]. Even worse, these complexes promotes abnormal disulfide bonding in cytoskeletal proteins such as actin, resulting in F-actin collapse [16, 18, 19]. Notably, glucose is the primary source of energy for the brain [20]. Previous studies suggested that reduced glucose levels in the brain are associated with neurological disorders [21, 22], with disulfidptosis playing a central role in the pathogenesis of these diseases [23, 24].

Increasing extracellular glucose levels does not inhibit seizures but rather increases the discharge frequency [25], potentially exacerbating cellular damage [26]. Indeed, exogenous glucose supplementation cannot address seizure-induced cell death, which may be related to glucose uptake mechanisms in neurons. Glucose transporter 3 (Glut3) on the neuronal surface can uptake glucose into cells and provide raw materials for energy metabolism [27, 28]. Down-regulating Glut3 expression impairs Glut3 transport, resulting in dysregulated glucose uptake by neurons and glucose deficiency-induced cell death [29, 30]. The transport of Glut3 from the cell to the membrane, i.e., Glut3 recycling, is dependent on the Rab11 pathway [29]. Rab11 is a marker for endosomal recirculation that regulates vesicle trafficking from the endosomal compartments and early endosomes to the trans-Golgi network and plasma membrane [31]. Rab11 mediates membrane-related transport processes, such as exocytosis and recirculation of membrane proteins to the plasma membrane [32]. Rab11 has multiple members, among which Rab11a is ubiquitous in multiple organs. Abnormal Rab11a function has been associated with seizures [33]. Nevertheless, the effect of Rab11a-mediated Glut3 recycling on seizure-induced neuronal disulfidptosis remains underexplored. Therefore, an in vitro model of seizure was constructed in the present study to evaluate the potential link between Glut3, disulfidptosis mechanisms and seizures by assessing cell survival, glucose levels, disulfidptosis biomarkers, Glut3 and Rab11a expression, and Glut3 recycling.

## Materials and methods

### Construction of the in *vitro* model of seizure

Neurons were harvested from newborn Sprague Dawley (SD) rats (24 h-old) purchased from the Animal Center of Guangxi Medical University. Primary neurons were cultured based on the methods outlined in a previous study [4]. The conditions for cell culture are as follows: 5% CO2 and 37 ℃ Two types of medium, serum-neurobasal medium and serum-free-neurobasal medium were used. The serum-neurobasal medium was used for the first 10 h of in vitro culture. After 10 h, the serum-neurobasal medium was removed and eplaced with a serum-free neurobasal medium. The serum-neurobasal medium was as follows: 88% GibcoDulbecco’s Modifed Eagle Medium: F-12(DMEM/ F12)(Gibco, A4192001), 1% Glutamax (Gibco, 35050061), 1% penicillin–streptomycin (Gibco, 15140–122) and 10% serum (Gibco, A3160902). The serum-neurobasal medium was as follows: 96% Neurobasal™-A (Gibco,10888022), 1% Glutamax (Gibco, 35050061), 1% penicillin–streptomycin (Gibco, 15,140–122) and 2% B27 supplement (Gibco, 17504044). On Day in vitro 15 (DIV-15), neurons were divided into a control (Ctrl) group and a magnesium-free (Mg^2+^-free) group and subsequently exposed to two different extracellular fluids (5% CO_2_, 37 ℃, 3 h). Specifically, neurons in the Ctrl group were exposed to normal extracellular fluid, and those in the Mg^2+^-free group were exposed to Mg^2+^-free extracellular fluid. The proportion of extracellular fluid was as follows: 145 mM sodium chlorid (NaCl),2.5 mM potassium chloride (KCl), 10mM HEPES, 2mM calcium chloride (CaCl_2_), 10mM glucose, 0.002mM glycine, and 1mM magnesium chloride (MgCl2). The proportion of Mg2+-free extracellular fluid was as follows: 145mM NaCl, 2.5mM KCl, 10mM HEPES, 2mM CaCl_2_, 10mM glucose, 0.002mM glycine.

Lentivirus (LV) was purchased from Sangon Biotechnology (Shanghai) Co., Ltd., including the LV negative control (NC) group (titer: 1 × 10^9^ TU/mL) and LV overexpressing Rab11a (OE) group (titer: 2 × 10^8^ TU/mL). To explore optimal multiplicity of infection (MOI), neurons in DIV-3 were exposed to LV with different MOI (MOI = 0, 1, 5, 10, 50, 100). After 24 h of exposure, the serum-free neurobasal medium containing the LV was replaced back into the normal serum-free neurobasal medium. On DIV-15, the neurons were used to measure cell survival and protein expression. After preliminary exploration, the best value of MOI was determined. Based on best value of MO, neurons (DIV-3) were transfected with LV. On DIV-15, LV-transfected neurons were exposed to Mg^2+^-free extracellular fluid.

The extracellular fluid, Mg^2+^-free extracellular fluid for the in vitro model, extracellular fluid, and intracellular fluid for action potential (AP) detection were prepared according to the methods established in our previous study [4]. The proportion of extracellular fluid (pH = 7.4) for AP detection was as follows: 122 mM NaCl, 2 mM KCl, 25mM HEPES, 2mM CaCl_2_, 4mM MgCl_2_, and 10mM glucose,. The proportion of intracellular fluid (pH = 7.3) for AP detection was as follows:110 mM KCl, 1mM NaCl, 2mM ethylene glycol tetraacetic acid (EGTA), 25mM HEPES, 4 mM adenosine 5′-triphosphate magnesium salt (Mg-ATP), 0.3 guanosine 5’-(disodium dihydrogen triphosphate) (Na2-GTP) and 10mM phosphocreatine. The resistance of patch electrodes was 3–6 MΩ. Digidata 1550 B patch-clamp amplifer and Axon Digidata 1550 B 16-bit data acquisition system were employed to obtain the APs (clamping voltage, -70 mV; mode, whole-cell recording). The pClamp 10.7 data acquisition software were utilized to analyze the data of APs.

### Glucose level measurement and cystine uptake assays

After lysis, glucose levels were determined in neurons using a glucose content assay kit (BC2500, Solarbio) [34, 35]. A Cystine Uptake Assay Kit was utilized to perform the cystine uptake assays. The Infinite m200 PRO (TECAN) was employed to measure glucose levels and cystine uptake.

### Analysis of neuronal survival rate

The 3-(4,5-dimethylthiazol-2-yl)-2,5-diphenyltetrazolium bromide (MTT) kit (Boster, AR1156) was used to measure cell survival rates. The Infinite m200 PRO (TECAN) was employed to measure MTT.

### NADP + and NADPH measurements

The NADP(H) Content Assay Kit (BC1105, Solarbio) was used to determine NADP + and NADPH levels [36]. The Infinite m200 PRO (TECAN) was employed to measure NADP + and NADPH levels. Finally, the ratio of NADP+ / NADPH was calculated.

### Western blot analysis

A protein extraction kit (Invent Biotechnologies, SD-001/SN-002; SM-005) was utilized to extract the total protein and surface protein of neurons. Western blot analysis was performed based on the methods described in previous studies [4, 30]. Primary antibodies used in the current study were anti- Glut3 antibody (Santa, sc-74399) (dilution: 1:500) [37], anti-xCT (xCT protein was coded by the SLC7A11gene) antibody (Abcam, ab307601, dilution: 1:500), anti-Rab11A antibody (Abcam, ab128913, dilution: 1:500) anti-GAPDH antibody (Sangon Biotech, D110016) (dilution: 1:10000), anti-alpha Tubulin1 antibody (abcam, ab7291, dilution: 1:10000) and anti-ATP1A1 antibody (Proteintech, 14418-1-AP) (dilution: 1:10000). The secondary antibodies were IRDye 800CW-conjugated goat anti-rabbit immunoglobulin IgG (product #5151, 1:2000, CST) and DyLightTM800-conjugated anti-mouse IgG (H + L) (product #5257, 1:5000; CST). Total proteins were normalized to internal reference proteins GAPDH or alpha Tubulin1 using Image J software. Surface proteins were normalized against ATP1A1.

### Colocalization assay

Colocalization staining of neurons was conducted according to the method outlined in a previous study [4]. Neurons were incubated with the first primary antibody, anti-Glut3 antibody (Santa, sc-74399) (dilution: 1:50) overnight at 4 ℃. After washing with PBS, they were incubated with a fluorescent secondary antibody (Boster, BA1101) (dilution: 1:100) (45 min, 37 ℃). Neurons were marked in green fluorescent. Thereafter, neurons were incubated with a second primary antibody, anti-Rab11A antibody (Abcam, ab316152) (dilution: 1:100), overnight at 4 ℃. After washing with PBS, they were incubated with a fluorescent secondary antibody (Boster, BA1090) (dilution: 1:100) (45 min, 37 ℃), with neurons marked in red fluorescent. Finally, the neurons were incubated with a DAPI staining solution for 3 min at room temperature. Fluorescence images of neurons were captured using a microscope (Olympus BX53).

### Co-immunoprecipitation (Co-IP) assay

Total natural proteins were extracted using the protein extraction kit (Invent Biotechnologies, SD-001/SN-002) [38]. To generate the antigen-antibody complex, the natural proteins were combined with anti-Glut3-antiboy (Santa, sc-74399) (overnight, 4 °C). CO-IP analysis was performed using the immunoprecipitation kit (Sangong Biotech, C600689). Protein samples were separated via SDS-PAGE and transferred to the NC membrane by wet transfer cells (Bio-Rad) (Merck Millipore Ltd.). Primary antibodies for western blot analysis were as follows: anti-Glut3 antibody (Santa, sc-74399) (dilution, 1:500) [37], anti-Rab11A antibody (Abcam, ab128913; dilution, 1:500).

#### Recycling assay for Glut3

The neurons were incubated at room temperature for 10 min with anti-Glut3 antibody (Santa, sc-74399) (dilution: 1:50). Next, they were washed with phosphate buffer (PBS) at 37 ℃ to remove unbound antibodies, and the neurons were cultured in an antibody-free medium at 37 ℃ for 20 min to induce the internalization of antibody-binding receptors. Subsequently, antibodies retained on the neuronal surface were incubated on ice for 4 min in 0.5 M of peel buffer (0.5 M NaCl and 0.2 M acetic acid). Then, neurons were washed with frozen PBS and incubated in a normal medium at 37 °C for 90 min to promote receptor recycling. The neurons were then immobilized with 4% polyformaldehyde and blocked with 5% bovine serum albumin. Glut3 recycled to the surface was detected by incubating neurons with a red fluorescent secondary antibody (Boster, BA1031). Neurons were permeabilized using 0.25% Triton X-100, and Glut3 was detected using a green fluorescent secondary antibody (Boster, BA1126). A microscope (Olympus BX53) was utilized to observe and capture fluorescence images of neurons. A total of 9 neurons in each group were used to calculate optical density. Recycling ratio = surface fluorescence signal/(surface signals + intracellular signals).

### Statistical analysis

The data are expressed as the mean ± standard deviation. Experimental data were compared between 2 groups using the independent sample *t* test. Experimental data were compared among groups (>2 groups) using one-way analysis of variance. SPSS 25.0 was used for the statistical analysis. A *p* value of < 0.05 was considered statistically significant.

## Results

### Seizures led to glucose deficiency and disulfidptosis in neurons

**Fig. 1:**
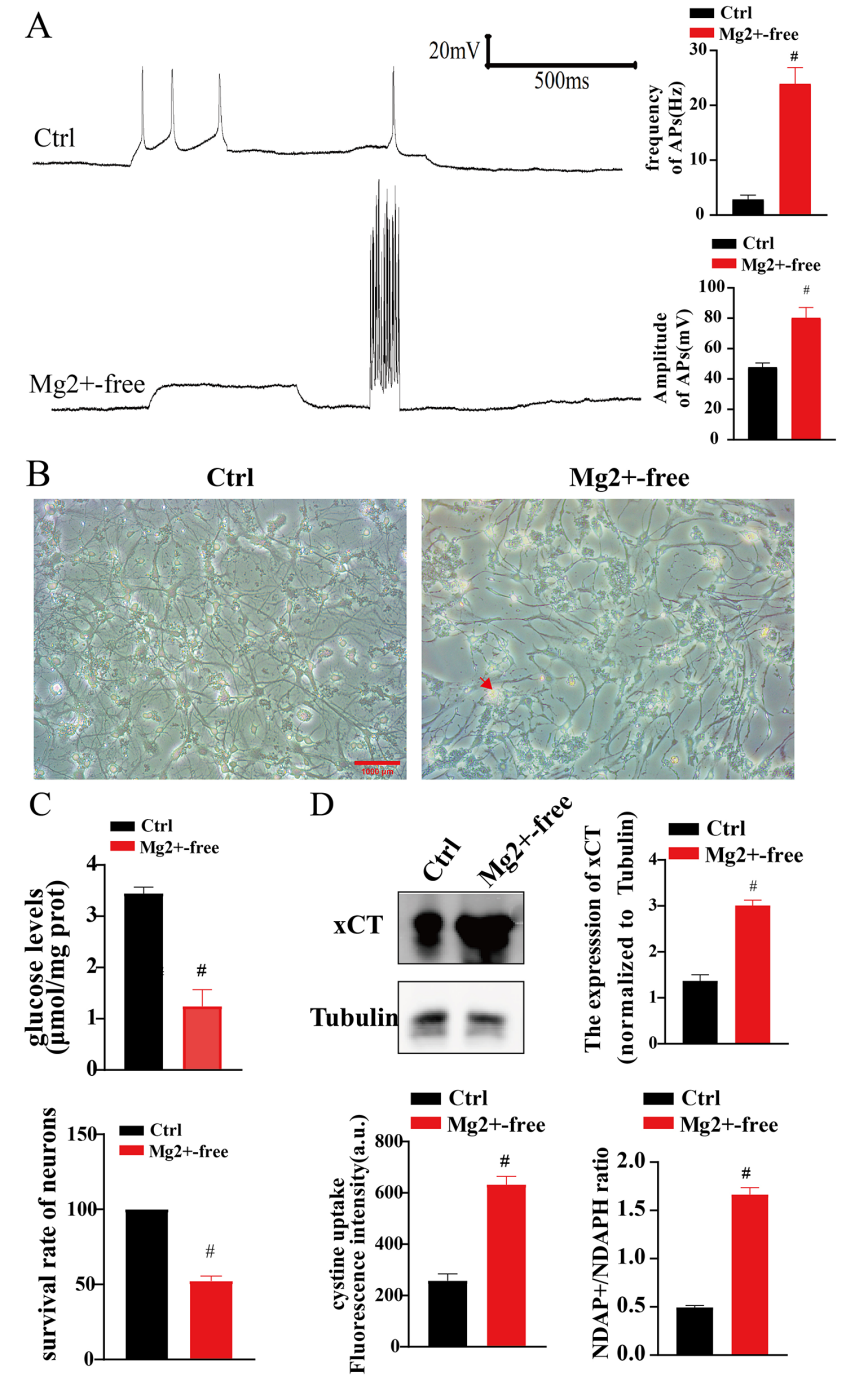
Seizures can lead to glucose deficiency and disulfidptosis in neurons. **A** The amplitude and frequency of neuronal APs were significantly higher in the Mg2+-free group (6 cells in each group;#, *p*＜0.01). **B** Neurons in the Ctrl group (×200): Neuronal bodies were full, with visible axons. Neurons in the Mg2+-free group were swollen and ruptured. Additionally, the number of neurons was significantly lower, with significant cell edema and level and no visible neural network structure. Red arrows indicate swollen and ruptured neurons. Scale: 100 μm. **C** Neuronal glucose levels were significantly lower in the Mg2+-free group. The survival rate of neurons in the Mg2+-free group was significantly lower (*n *= 6 in each group;*p＜0.05,#,*p*＜0.01). **D** xCT expression, the NADP/NADPH ratio, and the level of Cys uptake were significantly increased (*n *= 6 in each group;#*p*＜0.01).

Neurons exposed to Mg^2+^-free extracellular fluid for 3 h produce synchronized firing, which is considered as an in vitro model of seizures [4]. After exposed to Mg^2+^-free extracellular fluid for 3 h, the neuronal APs were detected by patch clamp. The amplitude and frequency of neuronal APs were significantly higher in the Mg^2+^-free group (*n* = 6 in each group; vs. Ctrl, *p*<0.01; Fig. 1A), which suggested the in vitro model of seizures was successfully constructed. The morphology of the neurons was then observed. In normal neurons, the cell bodies are round and full, showing a round or oval shape, the neurites are obvious, and the neurons are connected into a clear neural network structure. The neurons in the Ctrl group was with same morphology as normal neurons, which had full cell bodies and prominent network structures. In contrast, the neurons in the Mg^2+^-free group were with significantly lower numbers, displaying significant cell edema and disorganized neural network structures (Fig. 1B). Meanwhile, the level of neuronal glucose in the Mg^2+^-free group was significantly lower compared to the control group (*n* = 6 in each group; vs. Ctrl, *p*<0.01; Fig. 1C). Besides, the results of the MTT assay revealed significantly lower survival rates of neurons in the Mg^2+^-free group compared to the control group (*n* = 6 in each group; vs. Ctrl, *p*<0.01; Fig. 1C). To determine whether disulfidptosis is involved in seizure-induced cell death, the disulfidptosis markers were explored. In the Mg^2+^-free group, alterations in the levels of disulfidptosis markers were noted. For instance, the expression level of xCT was significantly higher in the Mg^2+^-free group compared to the control group (*n* = 6 in each group; vs. Ctrl, *p*<0.01; Fig. 1D). Likewise, the NADP/NADPH ratio was significantly higher (*n* = 6 in each group; vs. Ctrl, *p*<0.01; Fig. 1D). Lastly, the level of Cyss uptake was significantly higher (*n* = 6 in each group; vs. Ctrl, *p*<0.01; Fig. 1D). Those results demonstrated that disulfidptosis may play a significant role in seizures.

### Relationship between the recycling ratio of Glut3 and the expression of Rab11a

**Fig. 2:**
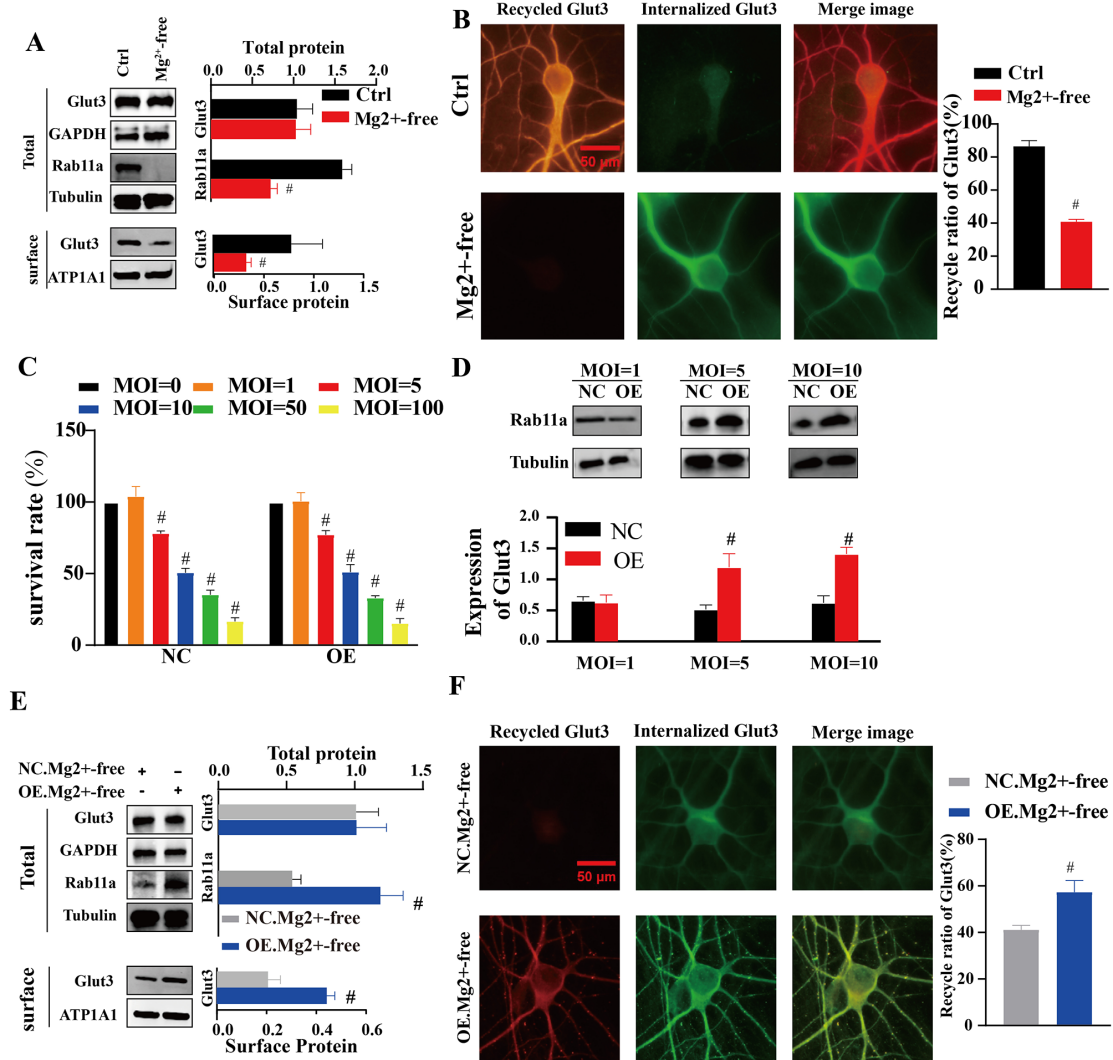
Relationship between the recycling ratio of Glut3 and Rab11a expression. **A** Total protein levels of Rab11a were lower in the Mg2+-free group. Surface Glut3 protein expression levels on neurons were lower in the Mg2+-free group (n=6 in each group;#p＜0.01).**B** The recycling ratio of Glut3 was lower in Mg2+-free group (9cells in each group; #p＜0.01)(×600, Scale: 50 μm). **C **The survival rate of neurons in different value of MOI between of NC group and OE gourp. The survival rate of neurons in NC group (n=6 in each group; #,vs MOI=0, p＜0.01). The The survival rate of neurons in OE group (n=6 in each group;#,vs MOI=0, p＜0.01). **D** The total protein expression level of Rab11 in different value of MOI between of NC group and OE group.In MOI=5 and MOI=10,the total protein expression level of Rab11a was higher in the OE group (n=6 in each group; #p＜0.01). **E** The total protein expression level of Rab11a was higher in the OE group. Surface Glut3 protein expression levels on neurons were higher in the OE group (n=6 in each group; #p＜0.01). **F** The recycling ratio of Glut3 was higher in the OE group (9 cells in each group; # p＜0.01)(×600, Scale: 50 μm).

The two proteins, Glut3 and Rab11a, were are involved in glucose metabolism in neurons [39, 40] and may be the key factors in disulfidptosis. To investigate the relationship between Glut3 and Rab11a in seizures, the expression of these two proteins were detected. On the one hand, the results of western blot analysis demonstrated that the total Glut3 protein levels were comparable between the Mg^2+^-free group and the control group (*n* = 6 in each group; vs. Ctrl, *p*>0.05, Fig. 2A). On the other hand, the total protein expression level of Rab11a was significantly lower in the Mg^2+^-free group (*n* = 6 in each group; vs. Ctrl, *p*<0.01, Fig. 2A). At the same time, the protein expression level of Glut3 on the neuronal surface was significantly lower in the Mg^2+^-free group compared to the control group (*n* = 6 in each group; vs. Ctrl, *p*<0.01, Fig. 2A). To further clarify the cause of reduced Glut3 on the neuronal surface, the recycling of Glut3 was analyzed. As anticipated, the recycling assay suggested that the recycling ratio of Glut3 was lower in the Mg^2+^-free group (9 cells in each group; vs. Ctrl, *p*<0.01, Fig. 2A). To investigate the potential link between Rab11a and Glut3, neurons were transfected with LV. In this experiment, the optimal value of MOI needs to be determined. Firstly, the effects of different MOI values of LV on the survival rate of neurons were examined. With MOI greater than 10, the survival rate of neurons was significantly reduced, even less than 50% (*n* = 6 in each group; vs. MOI = 0, *p*<0.01, Fig. 2C). Since high concentrations of LV(MOI = 50,MOI = 100) can lead to excessive neuronal mortality, these two groups of LV-transfected neurons were excluded. Subsequently, the transfected neurons were measured for Rab11a expression to determine the optimal MOI value. The expression of Rab11a was most significantly increased in MOI = 5 group and MOI = 10 group (*n* = 6 in each group; vs. MOI = 0, *p*<0.05, Fig. 2D). Since the survival rate of neurons in the MOI = 5 group was significantly higher than that of neurons in the MOI = 10 group, MOI = 5 was the optimal MOI value. After transfection with LV (MOI = 5), neurons in both the NC group and OE group were exposed to Mg^2+^-free conditions. The total protein expression level of Glut3 was similar between the OE and NC groups (*n* = 6 in each group; vs. NC, *p*>0.05, Fig. 2E). Conversely, the total protein expression level of Rab11a was higher in the OE group (*n* = 6 in each group, vs. NC, *p*<0.01, Fig. 2E). Similarly, the protein expression level of Glut3 on the neuronal surface was higher in the OE group compared to the NC group (*n* = 6 in each group, vs. NC, *p*<0.01, Fig. 2E). Overall, the recycling assay indicated that the recycling ratio of Glut3 was higher in the OE group (9 cells in each group vs. Ctrl, *p*<0.01, Fig. 2F).

### Interaction between Glut3 and Rab11a

**Fig. 3:**
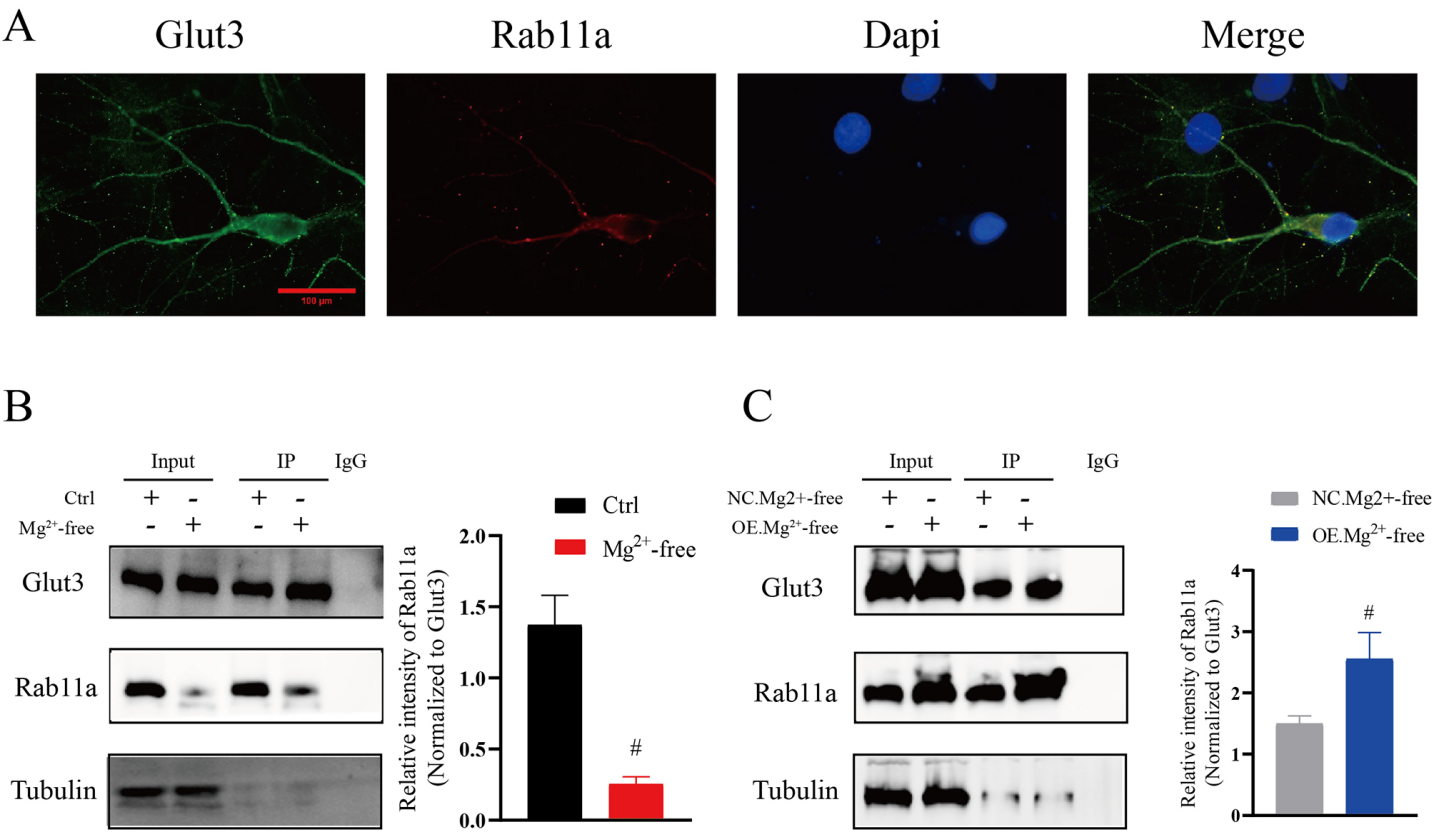
Interactions between Glut3 and Rab11a. **A** Glut3 and Rab11a were both expressed on neurons (×600, Scale: 100 μm). **B** The number of Glut3-Rab11a protein complex was decreased in the Mg2+-free group (*n* = 6 in each group; # *p*<0.01). **C** The number of Glut3-Rab11a protein complex was increased in the OE group (*n* = 6 in each group;#*p*<0.01)

To investigate the relationship between Glut3 and Rab11a, colocalization analyses were performed. The results showed that Glut3 and Rab11a were both expressed on neurons (Fig. 3A), signaling a colocalization relationship between both proteins. The CO-IP assay indicated that the number of Glut3-Rab11a protein complex was significantly lower in the Mg^2+^-free group compared to the control group (*n* = 6 in each group; vs. Ctrl, *p*<0.01, Fig. 3B), whereas the number of Glut3-Rab11a protein complex was higher in the OE group (*n* = 6 in each group; vs. NC, *p*<0.01, Fig. 3C).

### Rab11 alleviated glucose deficiency and inhibited seizure-induced neuronal disulfidptosis

**Fig. 4:**
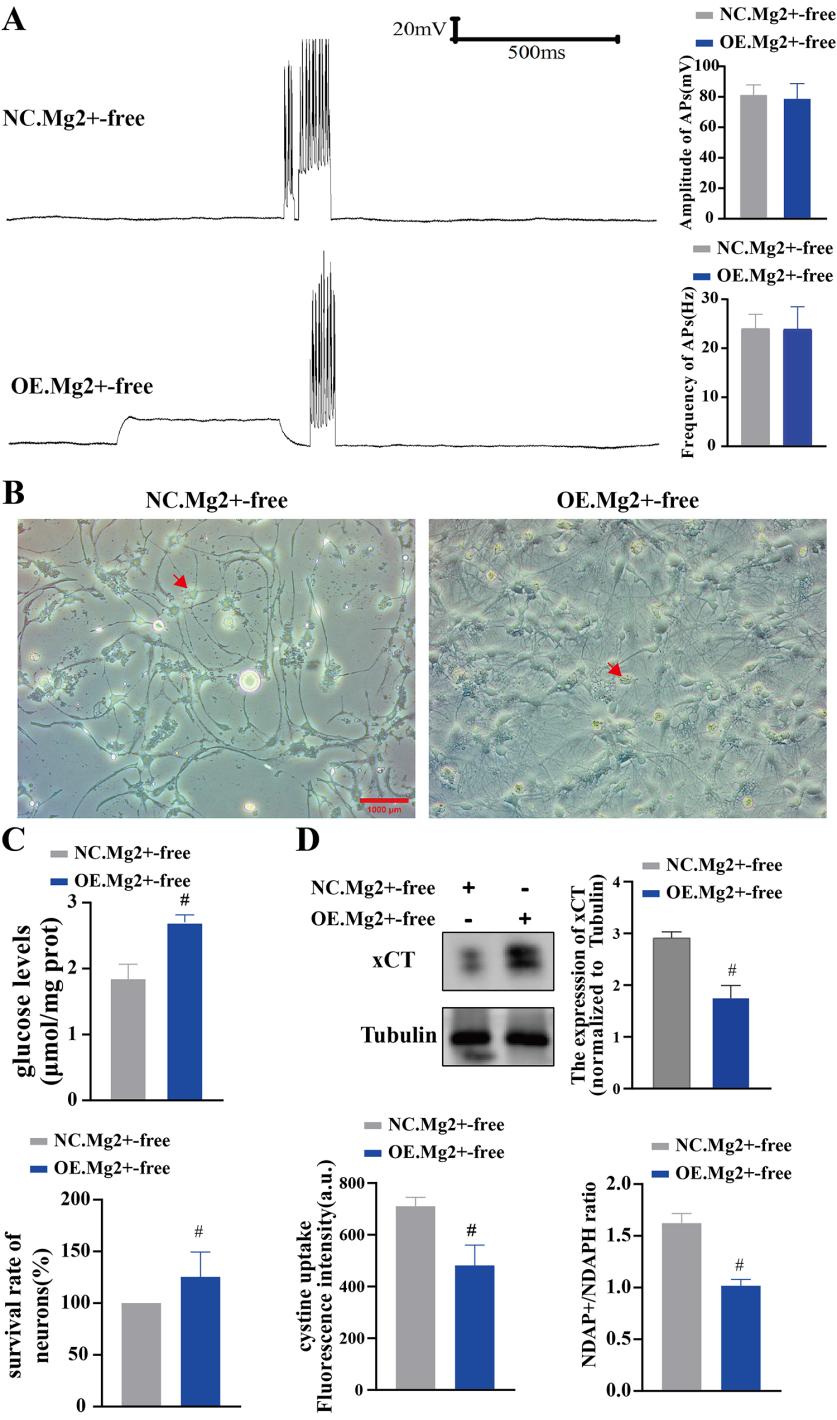
Rab11 alleviated glucose deficiency and inhibited seizure-induced neuronal disulfidptosis. **A** After exposure to Mg2+-free for 3 h, the amplitude and frequency of APs were comparable between the NC and OE groups. **B** Neurons in the NC group (×200): the number of neurons was significantly reduced, with significant cell edema and level and no evidence of a neural network structure. Neurons in the OE group (×200): the neuronal bodies were full, with visible axons and the presence of a network structure. Red arrows represent swollen and ruptured neurons. Scale: 100 μm. **C** Neuronal glucose levels were significantly higher in the OE group, whereas the survival rate of neurons was significantly lower (*n* = 6 in each group; #,*p*<0.01). **D** xCT expression, the NADP/NADPH ratio, and the level of Cys uptake were significantly decreased (*n* = 6 in each group; #*p*<0.01)

To clarify whether Rab11a could inhibit seizure-induced neuronal disulfidptosis and protect neurons, neuronal survival and disulfidptosis markers were again explored. After exposure to a Mg^2+^-free medium for 3 h, neuronal synchronous discharges were observed in both the NC and OE groups. Nevertheless, differences in the amplitude and frequency of APs between the NC group and the OE group were non-significant (*n* = 6 in each group; vs. NC, *p*>0.05, Fig. 4A). In the NC group, there were fewer neurons with round or oval bodies and obvious neurites. These neurons were observed with significant cell edema and the disorder of neural network structures. On the contrary, there were lots of neurons with round or oval bodies and obvious neurites in OE gorup. These neurons were observed with full bodies and obvious network structures (Fig. 4B). Moreover, the glucose level of neurons was significantly higher in the OE group compared to the NC group (*n* = 6 in each group; vs. NC, *p*<0.01, Fig. 4C). In addition, the results of the MTT assay showed that the survival rate of neurons was significantly higher in the OE group compared to the NC group (*n* = 6 in each group; vs. NC, *p*<0.05, Fig. 4C). Compared with the NC group, the expression level of xCT was significantly lower in the OE group compared to the NC group (*n* = 6 in each group; vs. NC, *p*<0.01, Fig. 4D). The NADP/NADPH ratio was significantly higher in the OE group (*n* = 6 in each group; vs. NC, *p*<0.01, Fig. 4C), whereas the level of cystine uptake was significantly lower (*n* = 6 in each group; vs. NC, *p*<0.01, Fig. 4C).

## Discussion

Seizures could lead to various serious consequences, including neuronal death, neuronal injury, and alteration of neuronal networks [41]. These consequences appear to be related to lower glucose metabolism, especially glucose deficiency [30, 42]. Seizures may result in lower glucose metabolism in the hippocampus on the ipsilateral side of the epileptic focus [10]. However, increasing extracellular glucose levels does not inhibit seizures but rather increases the discharge frequency [25], potentially exacerbating cellular damage [26]. Previous studies have shown that seizures inhibit cellular glucose uptake [43, 44], thereby reducing cellular glucose levels [45], which may account for exacerbating seizures by elevated extracellular glucose levels. Therefore, elucidating the causes underlying impaired glucose uptake by neurons may assist in unraveling seizure-induced neuronal glucose deficiency. Neurons primarily uptake glucose through Glut3, which is located on the neuronal surface [46]. Glut3, encoded by the Slc2a3 gene, contains 12 transmembrane helices comprising a regulated central hydrophilic pore and intracellular N- and C-terminal ends [47]. The low expression of Glut3 is considered as the potential pathogenesis of Alzheimer’s disease [48]. However, the relationship between Glut3 and seizures is not clear. In our study, based on the in vitro model of seizures, no significant change in the expression level of Glut3 was observed in neurons, the level of Glut3 on the neuronal surface was significantly decreased. These result indicated that the reduced Glut3 on neuronal surface may be associated with the impaired glucose uptake and seizure-induced neuronal glucose deficiency. Glut3 recycling is considered as be a key factor affecting the amount of Glut3 on the neuronal surface [29]. To investigate the the effects of seizures on Glut3 recycling, the recycling assay was performed. The result showed that the seizures could limit the Glut3 recycling. Whereas, the underlying mechanism of Glut3 recycling in seizures remains unclear.

The recycling of the neuronal Glut3 is mediated by the Rab11 29. Rab11a, the member of the Rab11, is ubiquitous in multiple organs [31]. Rab11a, the a recycling endosome regulator, could regulate the recycling of some proteins in neurons [49–51]. Previous studies demonstrated that the deficiency of Rab11a was the pathogenesis of Huntington’s disease [29, 40]. Hamdan et al. suggested that abnormal Rab11a function has been associated with seizures [33]. We found the expression of Rab11a was decreased in seizures, suggesting the Rab11a may play a key role in the underlying mechanism associated with seizures. To further elucidate the role of Rab11a in the regulation of Glut3 in seizures, neurons were transfected with LV to up-regulate the expression of Rab11a. These neurons overexpressing Rab11a were subsequently exposed to Mg^2+^-free fluid, and the expression of Glut3 on the surface and the recycling ratio of Glut3 were increased. These results conjointly suggested that Rab11a may up-regulate Glut3 levels on neuronal surfaces by promoting Glut3 recycling. More importantly, it was shown that Rab11a can promote protein transport by binding to proteins [31]. Furthermore, colocalization and CO-IP assays displayed the interaction between Rab11a and Glut3. In the in vitro model of seizures, the number of protein complex formed by Glut3 and Rab11a was decreased, whilst the number of Glut3-Rab11a protein complexes was higher in neurons overexpressing Rab11a. Hence, we concluded that Rab11a promoted the recycling of Glut3 through binding to Glut3, and seizures could inhibit the recycling of Glut3 by down-regulating the expression of Rab11a and reducing the the number of Glut3-Rab11a protein complexes.

Glucose deficiency induced by seizures can cause neuronal death, which in turn can exacerbate seizures [26]. Neurons undergo programmed death after seizure, including necrosis, apoptosis, pyroptosis and ferroptosis [52–55]. Liu et al. found a novel cell death mechanism, disulfidptosis. Interestingly, this new type of cell death could not be inhibited by ferroptosis, apoptosis, necroptosis and autophagy inhibitors [12]. Disulfidptosis is characterized by the abnormal accumulation of Cyss (one disulfide) caused by glucose deficiency and ameliorating glucose deficiency effectively inhibits disulfidptosis [12]. Previous studies have concluded that seizures can decrease the levels of cellular glucose [45], thereby stimulating disulfidptosis [13–16]. However, the potential relationship between seizures and disulfidptosis should be investigated further. We found that the glucose level and cell survival rate of neurons were significantly decreased in the in vitro model of seizures. Then, the levels of disulfidptosis markers were measured. The results unveiled that the expression level of xCT, NADP/NADPH ratio, and Cyss uptake level were increased, indicating that seizures led to glucose deficiency in neurons, depletion of NADPH, and an imbalance in the NADP/NADPH ratio, resulting in the inability of neurons to effectively clear Cyss. Disulfidptosis is a complex cell death mechanism that also involves mitochondrial respiratory chain complex I which also plays a vital role in NADP/NADPH metabolism [56–58]. In addition, xCT overexpression drove Cyss uptake by neurons, which may exacerbate disulfidptosis [16, 18, 19]. These factors collectively resulted in the excessive accumulation of disulfide and induced disulfidptosis, in which glucose appears to play an important role [13–16]. Maintaining glucose levels in neurons may be critical for inhibiting disulfidptosis. Interestingly, the glucose level and cell survival rate were higher in neurons overexpressing Rab11a. Besides, xCT expression, NADP/NADPH ratio, and Cyss uptake were lower. Overall, these results suggest that Rab11a can inhibit seizure-induced neuronal disulfidptosis to improve the neuronal survival rate.

Taken together, Rab11a can increase the number of Glut3-Rab11a protein complexes, thus facilitating Glut3 recycling and increasing Glut3 expression on the cell membrane. The up-regulation of Glut3 on the surface promotes neuronal glucose uptake, minimizes NADPH consumption, maintains the NADP/NADPH balance, and thus allows neurons to effectively clear Cyss. At the same time, the expression of xCT was down-regulated, which prevented neurons from uptaking Cyss, reduced the accumulation of disulfide in cells, and finally inhibited disulfidptosis. However, overexpression of Rab11a in neurons did not inhibit synchronous discharge. Navchaa Gombodorj et al.. suggested that Rab11a could promote cell proliferation [59]. Aberrant Rab11a-dependent trafficking of the neuronal surface protein may causes cell death [60]. Therefore, Rab11a in neurons may play the roles in improving neuronal survival and protecting neurons.

Neuronal death induced by seizures may result in cognition impairment [61], drug-resistant epilepsy [62], depression [63], etc. What’s worse, neuronal death leads to abnormal neural networks and hippocampal sclerosis, which could exacerbate the severity of epilepsy and increases the frequency of seizures [64–66]. Protecting the neurons could attenuate the severity of epilepsy and decrease the frequency of seizures [67, 68], improving the prognosis and life quality of individuals with epilepsy. Our study revealed that Rab11a may be a target for treating epilepsy and protecting neurons. Nevertheless, some limitations of this study cannot be overlooked. To begin, this study was exclusively based on an in vitro model, and the results were not validated in in vivo models. Future studies that construct animal models are warranted. Secondly, the sequence of changes in xCT expression, NADPH levels, and Cyss uptake during seizures were not assessed in this study. Finally, the role of the mitochondrial complex in seizure-induced disulfidptosis necessitates further research.

## Conclusion

In conclusion, our results corroborated that Rab11a-dependent recycling of Glut3 inhibited seizure-induced neuronal disulfidptosis by alleviating glucose deficiency, which may be a novel target for the treatment of epilepsy and neuroprotective strategies in the future.

## Electronic supplementary material

Below is the link to the electronic supplementary material.

Supplementary Material 1

## Data Availability

The datasets used and analysed during the current study are available from the corresponding author upon reasonable request.
