# Supplementary figures and images for: Rab11a-dependent recycling of Glut3 inhibits seizure-induced neuronal disulfidptosis by alleviating glucose deficiency
Source: Cell Biosci. 2025 May 28;15:69. doi: 10.1186/s13578-025-01396-9 (PMC12121293; doi:10.1186/s13578-025-01396-9)

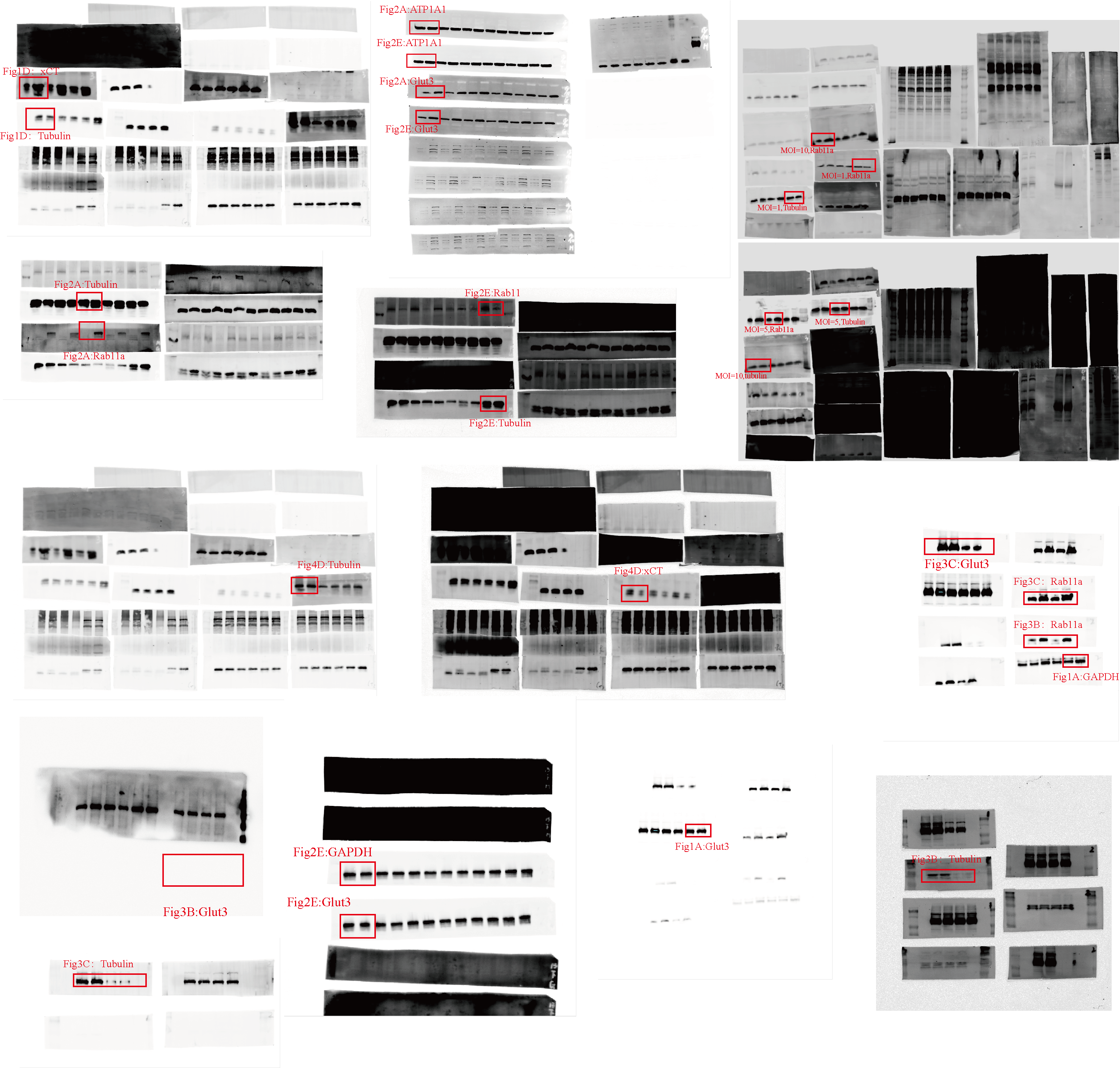

Supplement: Supplementary file 1 — Supplementary Material 1 [file 13578_2025_1396_MOESM1_ESM.tif]
